# Renalase Potential as a Marker and Therapeutic Target in Chronic Kidney Disease

**DOI:** 10.3390/biomedicines12081715

**Published:** 2024-08-01

**Authors:** Larisa Florina Serban-Feier, Elena Cuiban, Elena Bianca Gogosoiu, Elena Stepan, Daniela Radulescu

**Affiliations:** 1 Department of Nephrology, Faculty of Medicine, Carol Davila University of Medicine and Pharmacy, 020021 Bucharest, Romania; larisa-florina.feier@drd.umfcd.ro (L.F.S.-F.); elena.nichita@drd.umfcd.ro (E.S.); daniela.radulescu@umfcd.ro (D.R.); 2 Department of Nephrology, Sfantul Ioan Clinical Emergency Hospital, 042122 Bucharest, Romania; gogosoiu.bianca@gmail.com

**Keywords:** renalase, hypertension, cardiovascular disease, chronic kidney disease

## Abstract

Hypertension and cardiovascular disease are prominent features of chronic kidney disease, and they are associated with premature mortality and progression toward end-stage kidney disease. Renalase, an enzyme secreted predominantly by the kidney and identified in 2005, seems to be one of the missing pieces in the puzzle of heart and kidney interaction in chronic kidney disease by lowering blood pressure and reducing the overactivity of sympathetic tone. This review aims to summarize evidence from clinical studies performed on subjects with CKD in order to explore the value of renalase as a marker and/or a therapeutic target in this disease.

## 1. Introduction

Chronic kidney disease (CKD) is an increasingly worldwide condition affecting more than 10% of the population and it is strongly associated with hypertension (HT), incident cardiovascular disease (CVD) and high mortality [1,2]. The pathogenesis of CVD in CKD is multifactorial. Besides classical factors like HT, diabetes, obesity, and dyslipidemia, several specific abnormalities explain the high prevalence of CVD in CKD: the activation of the renin–angiotensin–aldosterone system, extracellular volume expansion, increased sympathetic activity, chronic inflammation, oxidative stress, mineral bone disease, accumulation of uremic toxins, etc. [3]. HT, left ventricular hypertrophy (LVH), coronary artery disease (CAD), peripheral artery disease (PAD), stroke, and heart failure (HF) with preserved or reduced ejection fraction are more prevalent in the CKD population, they are noted from early stages, and their incidence increases in parallel with decreasing GFR and increasing albuminuria [3]. By far, the most severe consequence of CVD in CKD is the increased risk of premature death: most CKD patients die of a cardiovascular or cerebrovascular complication before reaching the final stages of CKD [4,5].

Extensive research is performed to find new modalities for reducing the risk and consequences of CVD in CKD [6,7]. Among these, targeting the overstimulation of sympathetic activity in CKD has been less explored [8]. The stimulation of sympathetic activity in CKD is proven to be present from early stages and becomes extremely high in chronic dialysis patients [8]. It is an important contributor not only to hypertension genesis but also to the progression of kidney disease, increased left ventricular mass, cardiovascular events, and cardiovascular and general mortality [8,9]. Numerous factors are involved in the stimulation of sympathetic activity in CKD, including increased activity of the renin–angiotensin–aldosterone system, direct central sympathetic activation by afferent renal nerves in diseased kidneys, decreased nitric oxide production, oxidative stress, and the accumulation of uremic toxins, which are the most known causes [8]. Nevertheless, there is proof that kidneys are the main source of sympathetic stimulation: patients with bilateral nephrectomy who are on maintenance dialysis have normal sympathetic tone, while chronic dialyzed individuals without nephrectomy and also normal-functioning renal transplant recipients with failing native kidneys left in place have increased sympathetic activity [10,11]. These data suggest that the healthy kidney may be a source of one or more protective factors against sympathetic stimulation. One of the studied molecules in this field is renalase, an enzyme produced mainly by kidneys that is supposed to decrease blood pressure (BP) and lower sympathetic tone. This review aims to summarize evidence from clinical studies performed on subjects with CKD in order to explore the value of renalase as a marker and/or a therapeutic target in this disease.

## 2. Renalase—A Brief Description

Renalase was identified for the first time in 2005 by researchers from the laboratory of Yale School of Medicine [12]. According to the discoverers, renalase is a unique type of enzyme, a 32 kDa flavin adenine dinucleotide-dependent amine oxidase that is secreted mainly by the kidneys into the blood where it catabolizes circulating catecholamines, and thus it is involved in the control of sympathetic activity [12]. Although the proximal renal tubules are the main site of secretion of renalase, several reports demonstrated that renalase expression is present also in the glomerulus, myocardium, adipose tissue, liver, peripheral and central nervous system, small intestine, and skeletal muscles [12,13]. 

According to the initial findings, renalase is secreted into the blood by the kidney as prorenalase, the inactive form of renalase. Both the secretion and activation of prorenalase are stimulated by an increase in the levels of circulating catecholamines or by hypertension [14,15]. The consequences of the degradation of catecholamines by renalase consist of a decrease in arterial pressure, the attenuation of cardiac contractility, and a decrease in heart rate [12,16,17]. This classical pathway of renalase action, sustained by numerous studies, is contested by some researchers who consider that the hypotensive and vasodilatory effects of renalase are not due to its ability to degrade circulating catecholamines [18,19] but are secondary either to the oxidation of 2- and 6-dihydroNAD(P) to β-NAD(P)^+^ and H_2_O_2_ [19,20,21] or to its binding to the specific membrane receptor in various target organs [22].

However, even if the exact mechanism of action of renalase is incompletely elucidated, an impressive number of reports in the literature provide evidence that renalase is an important player in the pathogenesis of HT [23,24,25] and several cardiometabolic conditions [26].

## 3. Renalase in Chronic Kidney Disease

The kidney plays a central role in the metabolism of renalase as it is the main source of plasma renalase secretion. Besides serum catecholamines, the levels of plasma renalase also depend on renal perfusion and renal function [15,17,27]. 

Since its discovery in 2005, renalase has been the subject of several studies in subjects with CKD, either humans or laboratory animals (Table 1 and Table 2). The objectives of these studies can be grouped into three categories: establishing the serum or tissue level of renalase in different stages of CKD (Table 1), analyzing the value of renalase as a biomarker in CKD (Table 1), and investigating the effect of the therapeutic administration of renalase in patients with CKD (Table 2).

Since the kidneys are the main source of renalase secretion into the plasma, it would be expected that its serum level would decrease in all situations with reduced functional renal mass, and, as a result, the level of circulating catecholamines would increase with the occurrence of all the deleterious effects of sympathetic overstimulation. Indeed, researchers who first identified renalase proved that, in a small sample of chronic hemodialysis patients, the plasma levels of renalase were almost undetectable [12]. In a further study on nephrectomized rats, it was revealed that renalase expression in the failing kidney is diminished and also that in CKD there is a deficient activation of prorenalase in active renalase despite increased level of circulating catecholamines [15]. Yet, in numerous subsequent clinical studies, renalase serum levels were reported as being increased in CKD in an inverse relationship with GFR, such as the highest levels are noted in anuric maintenance dialysis patients [28,29,30,31] (Table 1). 

Also, in a study performed by Yilmaz R et al., it was revealed that, after transplantation, serum renalase levels increased in donors and decreased in recipients [25]. This discrepancy may be explained by a lack of standardization of the method used for measuring circulating renalase and/or by the fact that renalase circulates in the blood in different forms [32,33]. In the initial study performed by the researchers who first identified renalase, the Western blotting analysis with a renalase-specific polyclonal antibody was utilized, while in the rest of the studies, commercial locally available ELISA kits with a monoclonal antibody were used. Two of the discoverers of renalase, namely Desir G and Peixoto AJ, put forward the idea that, as kidney function diminishes in CKD, there is the possibility of the progressive accumulation of more and more renalase multimers that react with some monoclonal antibodies used in ELISA assays [33]. They reinforced this explanation by the fact that, in individuals with normal kidney function, the two available tests give almost identical results [32,33]. Other explanations offered by the literature refer to increased accumulation of renalase or renalase metabolites as residual renal function (RRF) diminishes, increased production of renalase from extrarenal sites as a reaction to increased catecholamines levels in CKD, or even the cross-reaction of the tests utilized with other substances in uremic blood [30,34]. However, as shown in Table 1, it is worth mentioning that, among the studies reporting use of the same commercially available ELISA kit, there are significant differences in the serum level of renalase for the same stage of pre-dialysis CKD [28,30,35] and even for healthy controls [29,30,35,36,37]. The same situation can be observed in maintenance dialysis patients [36,38], but in these cases, the differences may be explained by the level of RRF (as renalase is a low-molecular-weight protein that may be freely excreted by the kidneys [31,36,39,40,41]), the moment of measuring the plasma level of renalase (as it is proven that renalase may be removed by dialysis [31]), and by the type of extracorporeal technique (as there is proof that hemodiafiltration may remove a higher amount of renalase from circulation [31]). It is also striking that the results are expressed either in ng/mL, µg/mL, or ng/L, probably as it is specified in the ELISA kits available in different countries.

In 2019, Desir G et al. reported the development of a new ELISA assay that utilizes the m-28 anti-renalase antibody that targets the region of renalase that binds to its cell-surface receptor and initiates intracellular signaling [42]. Using this highly specific anti-renalase antibody and performing a special treatment of the plasma sample that exposes the binding sites of the m-28 antibody, Desir and his collaborators measured, in pre-dialysis CKD patients, the total renalase and found that its serum levels decreased as GFR decreased. Using untreated plasma, they measured only renalase with naturally exposed sites for the m-28 antibody (i.e., active renalase); they called it free renalase, and they found that it represented only a small percentage from total renalase which, in fact, increased as GFR decreased [42]. Thus, the researchers concluded that further studies are needed to understand the balance between free and total renalase in CKD.

Regarding the potential of renalase as a biomarker in CKD, studies that have been performed to date are promising in the field of prediction of CVD and/or the progression of CKD (Table 1). 

Numerous studies prove that renalase is involved in the modulation of cardiac function in CKD. Increased levels of serum renalase were reported to be associated with increased prevalence and/or severity of hypertension and left ventricular hypertrophy in pre-dialysis CKD patients [25,28,30,43,44,45,46]. Statistical analysis in some studies proved that increased plasma renalase in CKD can be used as a marker for the prediction of the occurrence of cardiovascular disease [28], hypertension [28], left ventricular hypertrophy [45,47], coronary artery disease [48], major adverse cardiovascular events [29], and cardiac and all-cause death [29,36,42,46]. Increased renalase levels were reported in some studies to have a synergistic effect with several already validated markers of CVD in CKD. In the study of Gluba-Brzózka A et al., CAD patients with CKD had significantly higher renalase, osteocalcin, matrix metalloproteinase-2 and tissue inhibitor of metalloproteinase-2 and lower fetuin-A than CAD patients without CKD [35]. Likewise, in a cohort of patients with CAD, Li YH et al. found that the combination of high serum renalase with CKD was a significant risk factor for increased serum endothelin-1 [48]. The authors speculated that increased renalase in CKD may increase circulating endothelin-1, and thus supplementary augments the risk for CAD in these patients. There are also studies proving a direct relationship between plasma renalase and pulse wave velocity [49], serum copeptin [45], or advanced oxidation protein products [50]. Nevertheless, there is a significant inconsistency in the findings across studies (Table 1), especially when comparing the results of pre-dialysis CKD patients with those of maintenance dialysis patients. No correlation between serum renalase levels and BP was present in most studies performed on maintenance dialysis patients [34,36,39,40]. Moreover, Malyszko J et al. found lower renalase levels in hypertensives than in normotensives [51]. Also, no correlations between serum renalase and catecholamines levels were found in dialysis patients [36,50], or even an inverse relationship was revealed [37]. These discrepancies may be related not only to the tests utilized for measuring renalase, as stipulated above, but also to RRF, the removal of both renalase and catecholamines by dialysis, the chronic activation of sympathetic activity, the interdialytic gain and the need for ultrafiltration, dialysis vintage, the presence of heart failure or other comorbidities, etc. [52,53]. Despite the heterogeneity of the results in clinical studies, several works prove an extraordinary potential of exogenous renalase administration (Table 2). Renalase infusion in rats in the original report was followed by a decrease in cardiac contractility, heart rate, and blood pressure and the prevention of a compensatory increase in peripheral vascular tone [12]. Similar effects were noted in further studies on laboratory animals with CKD, but, more than that, the researchers demonstrated the ability of renalase to partially reverse LVH and cardiac remodeling in CKD by attenuating cardiomyocyte hypertrophy and interstitial myocardial fibrosis [17,54,55].

As previously mentioned, most studies found an inverse relationship of renalase serum levels with eGFR in pre-dialysis CKD or RRF in maintenance dialysis patients (Table 1). Although the ranges of serum renalase for each stage of CKD have not been established, the usefulness of renalase as a marker of CKD progression is reported by some studies. In a median follow-up of 56 months considering patients with CKD stages 3–4, Baek SH et al. demonstrated that each 10 μg/mL increase in serum renalase was associated with significantly greater hazards of all-cause mortality and adverse renal outcomes [46]. In a cohort of kidney-transplanted patients, Stojanovic D et al. revealed that renalase was a strong predictor of early graft dysfunction in both univariate and multivariate analyses [56]. The direct involvement of renalase in CKD progression has been suggested in several works. Analyzing renal tissue samples in a cohort of biopsied patients with chronic nephropathies, Huang YS et al. proved that the expression of renalase was inversely correlated with the renal tubule injury index and the tubular epithelial cell apoptosis index [57]. As Bcl-2 expression was positively correlated with the expression of renalase, the researchers put forward the idea that renalase might reduce the apoptosis of renal tubular epithelial cells through the mitochondrial apoptosis pathway. By increasing the renal expression of renalase in rats with established tubulointerstitial fibrosis, Wu Y et al. found in two consecutive reports that renalase attenuates fibrosis through the suppression of tubular epithelial–mesenchymal transition through the inhibition of the ERK (extracellular-regulated protein kinase) pathway [58], and attenuation of oxidative stress [59]. The nephroprotective potential of renalase is also proved by the study of Yin J et al. on 5/6 nephrectomized rats; after 4 weeks of intravenous treatment with renalase, a significant decrease in proteinuria and blood urea nitrogen as well as a significant increase in creatinine clearance were observed [54]. Analyzing renal tissue samples in animals treated for 4 weeks with renalase, the authors found a significant reduction in glomerular hypertrophy and interstitial fibrosis, changes associated with a concomitant decrease in the mRNA expression of fibrosis markers, pro-inflammatory cytokines, and NADPH oxidase components [54]. Two studies performed on mice suggest that renalase administered before an ischemic or a nephrotoxic insult may prevent acute kidney injury (AKI) or chronic kidney disease by improving the viability of proximal tubules through the inhibition of necrosis, apoptosis, and inflammation in the renal tubules [60,61]. In both studies, the authors found that renalase-deficient mice developed more severe forms of AKI or CKD.

In conclusion, experiments on laboratory animals demonstrate that treatment with native or recombinant renalase could become a valuable therapeutic tool for preventing and mitigating the progression of CKD and CVD in CKD. However, besides the fact that it has not been used so far on human subjects, there are many uncertainties regarding the time of administration and the effective doses. In the study in which they demonstrated that treatment with renalase can prevent ischemic AKI [60], the authors observed that the renoprotective effect of renalase is manifested only if it is administered early, in the first 30 min after reperfusion, but not later; as a result, they suggested that renalase could be administered to patients at high risk of ischemic AKI. Also, the authors observed that nephroprotection is obtained only in the case of the administration of lower doses of renalase; they speculated that at high doses, the BP-lowering effect of renalase would be followed by negative circulatory consequences that would nullify its nephroprotective effects [60].

**Table 1:** Studies on renalase in human subjects with chronic kidney disease.

Study, Year	Patients	Aim	Significant Results	Assay for Serum RNLS (Unit of Measure)	Formula for CKD Staging	Serum Levels of RNLS
**Xu, 2005 [12]**	8 HD, 4 healthy	Identification of a new protein secreted by the kidney	RNLS almost undetectable in HD pts	Western blot	-	-
**Zbroch E,** **2012 [43]**	62 transplanted 27 healthy controls	Associations between RNLS, BP, and kidney function	RNLS higher in transplanted pts than in controls, higher in hypertensives RNLS inverse correlation with eGFR.	ELISA* (µg/mL)	MDRD; CKD-EPI; Clcr	Transplanted: 6.72 ± 2.86; Controls: 3.86 ± 0.73
**Zbroch E,** **2012** **[39]**	104 HD 27 healthy controls	RNLS levels in HD pts and relationship to BP control, type of antihypertensives, RRF	RNLS higher in HD than controls inverse relationship between RNLS and RRF No correlation between RNLS and BP, HR, HD vintage, or type of treatment.	ELISA* (µg/mL)	-	HD: 27.53 ± 7.18; Controls: 3.86 ± 0.73
**Zbroch E,** **2012** **[40]**	26 PD 27 healthy controls	RNLS levels in PD pts and relationship to BP control, type of antihypertensives, RRF, PD parameters	RNLS higher in PD pts than in controls; RNLS directly correlated with dialysis vintage and inversely with RRF No relation between RNLS and BP, antihypertensive treatment type, dialysis dose, or PET.	ELISA* (µg/mL)	-	PD: 19.24 ± 4.50; Controls: 3.86 ± 0.73
**Malyszko, 2012** **[51]**	34 HD patients 22 healthy controls	Relation between RNLS, BP, stroke, and CV status	RNLS higher in HD than controls; In HD, RNLS lower in pts with history of stroke, lower in hypertensive than in normotensive, higher in glomerulonephritis vs. other etiologies.	ELISA* (µg/mL)	-	HD: 17.51 ± 6.73; Controls: 3.99 ± 1.73
**Zbroch E, 2013** **[36]**	76 HD 26 PD 27 healthy controls	Relation between RNLS and CA level, HR, BP, type of antihypertensives, and RRF	RNLS higher in HD vs. PD; No correlation between CA, RNLS, and BP; RNLS correlated directly with dialysis vintage and inversely with RRF; Higher RNLS levels in patients with cardiac death.	ELISA* (µg/mL)	-	Dialyzed: 25.37 ± 7.3; Healthy: 3.86 ± 0.74
**Gluba-Brzózka, 2014** **[35]**	139 CKD pts 45 subjects with normal kidney function	Describing markers of increased risk of CAD in CKD	Decreased levels of fetuin-A and increased concentration of osteocalcin, RNLS, MMP-2, and TIMP-2 in CKD pts with CAD RNLS correlated with serum calcium and phosphorus.	ELISA* (ng/mL)	MDRD	Controls: 251.0 ± 157.0; CKD stages 1 + 2: 354.5 ± 181.6; 3: 235.3 ± 152.3; 4: 279.4 ± 162.2; 5: 369.0 ± 110.5
**Wang F, 2015** **[30]**	87 CKD stages 1–5, including maintenance dialysis	Serum levels of RNLS and CA and their relationship with other renal function indicators	Serum levels of RNLS and RNLS/CA ratios higher in CKD 3–5 than CKD stage 1–2. RNLS no difference between CKD stage 1–2 and controls Serum RNLS highly correlated with CA and systolic BP.	ELISA* (ng/L)	MDRD	Controls: 167.8 ± 69.4; CKD 1: 169.4 ± 31.9 CKD 2: 159.0 ± 43.5 CKD 3: 226.6 ± 120.0 CKD 4: 286.9 ± 157.0 CKD 5:198.6 ± 76.2 CKD 5-ND: 242.2 ± 67.8 CKD 5-HD: 169.4 ± 58.8 CKD 5-PD: 232.9 ± 93.9
**Malyszko J,** **2015** **[31]**	100 HD pts 17 HDF pts 24 healthy controls	RNLS concentration in serum, urine, and ultrafiltrate	Higher removal of RNLS in HDF vs. HD Direct correlation between residual urine and urine RNLS and inverse with serum RNLS Patients with bilateral nephrectomy had the highest serum RNLS.	ELISA* Western blot	-	-
**Cerqueira A, 2015** **[62]**	64 pre-dialysis CKD pts	Association between plasma RNLS, renal function, and markers of endothelial dysfunction	Increased plasma RNLS correlated with the decrease of eGFR.	ELISA*	Not stipulated	Not available
**Stojanovic D, 2015** **[56]**	73 renal transplant recipients 32 healthy controls	Relation between renal dysfunction, RNLS, and endothelial dysfunction parameters	RNLS was a strong predictor of decreased GFR Endothelial nitric oxide synthase was a strong protective factor for kidney function.	ELISA* (ng/mL)	Cockcroft-Gault	Transplant: 141.82 ± 36.47 Healthy: 16.36 ± 4.13
**Oguz EG, 2016** **[38]**	50 HD, 35 healthy controls	Relation between RNLS and LVH	There was no significant association of RNLS with LVMI; RNLS higher in HD than in healthy; positive correlation with creatinine and dialysis vintage.	ELISA* (ng/mL)	-	HD: 212 ± 127; Controls: 116 ± 67
**Zbroch E, 2016** **[63]**	211 CKD pts stages 1–5 with HT	Impact of age on RNLS and CA and relation to BP control and CVD	Higher RNLS and dopamine in pts >65 years; No relation between RNLS and echocardiographic abnormalities or type of treatment.	ELISA* (µg/mL)	Not stipulated	>65 years: 20.59 (1.9–62) <65 years: 13.14 (0.05–49.9)
**Gok Oguz E, 2017** **[41]**	40 PD, 40 healthy controls	Serum level of RNLS and relation with EAT thickness.	RNLS higher in PD than in healthy; positive correlation with CRP, inverse correlation with RRF; no correlation with EAT.	ELISA* (ng/mL)	-	PD: 176.5 (100–278.3) Healthy: 122 (53.3–170.0)
**Dziedzic M, 2017** **[50]**	50 HD	Relation between serum RNLS and AOPP	No significant correlations between CA and RNLS; positive correlation between RNLS and AOPP.	ELISA* (μg/mL)	-	44.8 ± 6.5
**Stojanovic D, 2017** **[64]**	73 renal transplant recipients, 32 controls	Relationships between serum RNLS and parameters of endothelial dysfunction, lipids, and GFR	RNLS inversely correlated with GFR, Hb, and WBC; directly correlated with creatinine and cholesterol; no correlation with parameters of endothelial dysfunction and age.	ELISA* (ng/mL)	Cockcroft-Gault	Transplanted: 141.82 ± 36.47 Healthy: 16.36 ± 4.13 ng/mL
**Huang YS, 2018** **[57]**	72 biopsies in pts with chronic nephropathies	Expression of RNL in renal tissue samples	Renal tubule injury index and tubular epithelial cell apoptosis index showed a negative linear correlation with RNLS.	Immunohistochemistry	-	-
**Skrzypczyk P, 2019** **[49]**	38 CKD 38 healthy controls	RNLS levels in children with CKD, correlation with BP and markers of endothelial stiffness	RNLS correlated with CKD stage and pulse wave velocity, no correlation with BP or age.	ELISA* (μg/mL)	Schwarz formula	CKD: 59.45 ± 23.25 Healthy: 27.20 ± 5.15
**Baek SH, 2019** **[46]**	383 patients with CKD; follow-up 56 months	Whether plasma RNLS is a biomarker for CKD progression, MACCEs, or mortality	Each 10 μg/mL increase in serum RNLS was associated with significantly greater hazards of all-cause mortality and adverse renal outcomes; RNLS not associated with the rate of MACCEs	ELISA** (μg/mL)	Cockcroft-Gault	CKD 3: 67.7 ± 29.6 CKD 4: 79.7 ± 36.4 Controls: 28.2 ± 5.1
**Li YH, 2018** **[48]**	342 non-diabetic with CAD	Relation between serum RNLS, CKD, and ET-1 levels in patients with CAD	ET-1 levels positively correlated with serum RNLS and inversely correlated with the eGFR; In multivariate analysis, the combination of high serum RNLS with CKD was a significant risk factor for increased serum ET-1 levels.	ELISA* (ng/mL)	MDRD	CKD: 46.8 ± 17.1 Non-CKD: 33.9 ± 9.9
**Al-Shamma ZAA, 2018** **[44]**	68 non-diabetic CKD stages 2–5 50 healthy controls	Level of RNLS in different stages of CKD and relation with HT	Progressive increase of RNLS as GFR decreases and as Cystatin C increases; no difference between CKD stages 2–3 and controls; positive relation of RNLS with systolic BP in CKD 5.	ELISA*** (ng/mL)	CKD-EPI Cystatin C	CKD 2–3: 52.4 ± 11.8 CKD 5: 103.4 ± 36.3 Controls: 48.8 ± 5.7
**Wiśniewska M, 2019** **[65]**	155 CKD 30 healthy	Level of RNLS in serum, urine, and erythrocytes	Increased RNLS level in serum and decreased level in erythrocytes in CKD; no differences in the urine level No influence of primary renal disease.	ELISA* (ng/mL)	CKD-EPI	CKD: 103 (55.6–166) Healthy: 17.7 (16.3–21.8)
**Desir G, 2019** **[42]**	267 CKD different stages	Association between renal function and plasma RNLS with a new developed ELISA method Association between plasma RNLS and mortality	Total RNLS correlated positively with GFR and negatively with CKD stages No correlation between GFR or CKD stages and free RNLS % free RNLS correlated negatively with GFR and positively with CKD stages % free RNLS independently predicted death at both 1 year and 5 years.	ELISA with monoclonal antibody m28-renalase (µg/mL)	CKD-EPI	Total RNLS: 4.1–49.4 Free RNLS: 0.13–2.6
**Serwin NM, 2020** **[66]**	62 pre-dialysis CKD 28 healthy	The balance between serum and urine RNLS in healthy and CKD	Serum RNLS and serum-to-urine ratio significantly higher in CKD No relation between serum RNLS and GFR or proteinuria Serum RNLS was the only significant positively associated with urinary RNLS.	ELISA**** (ng/mL)	MDRD	CKD 1: 34.73 (20.71–129.7) CKD 2: 41 (18.11–104) CKD 3: 32.29 (14.15–106.35) CKD 4: 37.67 (26.6–85.77) Healthy: 11.1 (2.5–26.5)
**Cerqueira A, 2021** **[28]**	40 pre-dialysis CKD, mean follow-up 65 months	Relationship between RNLS and CV and renal outcomes	Significant higher RNLS in stages 4 + 5 vs. stages 1 + 2 RNLS predicted CKD progression, CVD, HT, diabetes, and dyslipidemia.	ELISA* (µg/mL)	CKD-EPI	Stages 1 + 2: 42.03 Stage 3: 71.35 Stages 4 + 5: 83.53
**Wisniewska M, 2021** **[37]**	77 HD, 30 healthy	RNLS levels in serum, erythrocytes, and urine	In HD, RNLS higher in serum and urine and lower in erythrocytes than in controls Plasma level of CA lower in HD vs. control.	ELISA* (ng/mL)	CKD-EPI	HD:185.5 ± 64.3 Controls: 19.6 ± 5.0
**Knop W, 2021** **[29]**	90 CKD stages 1–5 including HD 30 healthy Median follow-up 18 months	Relation between serum RNLS and occurrence of MACE, all-cause mortality, and the need for dialysis initiation	RNLS higher in HD vs. other stages or vs. controls; no difference between whole CKD and controls RNLS correlated only with mortality in HD RNLS correlated with MACE occurrence and all-cause deaths in all groups of CKD patients.	ELISA**** (µg/mL)	CKD-EPI	CKD: 22.5 (19.9–25.1) Controls: 23.9 (19.4–25.9)
**Wiśniewska M, 2021** **[34]**	27 HD with bilateral nephrectomy (BN); 46 HD anuric without nephrectomy; 30 healthy with normal kidney function	The effect of bilateral nephrectomy on RNLS and CA levels in the serum and erythrocytes	In the post bilateral nephrectomy HD, serum RNLS was the lowest, and erythrocytes’ RNLS was the highest. In both HD groups, no correlation between serum and erythrocytes RNLS and BP; in controls, direct relationship between RNLS and BP	ELISA**** (ng/mL)	Serum creatinine	HD with BN: 101.1 ± 65.5 Healthy: 19.6 ± 5.0 HD anuric without nephrectomy: 177.2 ± 68.3
**Yilmaz R, 2021** **[25]**	20 kidney donors (with unilateral nephrectomy) 20 kidney recipients	The effect of kidney transplantation on RNLS and relation to BP	Before transplantation, RNLS lower in donors and higher in ESRD pts After transplantation, serum RNLS increased in donors and decreased in recipients RNLS associated with change in MAP and circadian rhythm of BP in donors and recipients.	ELISA***** (µg/mL)	Creatinine clearance	Kidney donors - Before KTx: 125.2 ± 35.0 - After Ktx: 140.2 ± 73.6 Kidney recipients - Before KTx: 242.4 ± 147.0 - After KtX: 162.3 ± 53.0
**Gamal SG, 2021** **[47]**	90 HD	Relation between plasma RNLS and LVMI	RNLS significantly increased with the moderately and severely abnormal LVMI.	ELISA (ng/mL) (assay kit not specified)	-	Cut-off for prediction abnormal LVMI > 57.9 (sensitivity 92.3%, specificity 84.0%)
**Kamel RA, 2022** **[45]**	40 HD with LVH 30 HD without LVH	Relation between plasma RNLS, LVMI, and copeptin	A positive correlation between serum RNLS and all of systolic, diastolic BP, duration of HD, LVMI, and serum copeptin.	ELISA* (ng/mL)	-	Cut-off for prediction LVH > 115 (sensitivity 92.5%, specificity 86.7%)

Legend. RNLS—renalase; CKD—chronic kidney disease; HD—hemodialysis; pts—patients; BP—blood pressure; eGFR—estimated glomerular filtration rate; ELISA—enzyme-linked immunosorbent assay; MDRD—modification of diet in renal disease formula; CKD-EPI—chronic kidney disease—epidemiology collaboration formula; Clcr—clearance of creatinine; RRF—residual renal function; HR—heart rate; PD—peritoneal dialysis; PET—peritoneal equilibration test; CV—cardiovascular; CA—catecholamines; CAD—coronary artery disease; MMP-2—matrix metalloproteinase-2; TIMP-2—tissue inhibitor of metalloproteinase-2; HDF—hemodiafiltration; LVH—left ventricular hypertrophy; LVMI—left ventricular mass index; CVD—cardiovascular disease; EAT—epicardial adipose tissue; CRP—C-reactive protein; AOPP—advanced oxidation protein products; Hb—hemoglobin; WBC—white blood cells; MACCEs—major adverse cardiac and cerebrovascular events; ET-1—endothelin-1; MACE—major adverse cardiovascular events; KTx—kidney transplantation; ELISA*—ELISA USCN Life Science Inc., Wuhan, China; ELISA**—ELISA Cloud-Clone Corp. (Katy, TX, USA); ELISA***—ELISA (Cusabio, Wuhan, China); ELISA****—ELISA kit (EIAab, Wuhan, China); ELISA*****—ELISA Kit (Eastbiopharm Co., Ltd., Hangzhou, China).

**Table 2:** Studies revealing the potential use of renalase in the treatment of chronic kidney disease.

Authors, Year	Patients	Aim	Significant Results
**Xu J, 2005** **[12]**	Rats	Effects of RNLS infusion	Decrease in cardiac contractility, HR, and BP and prevention of compensatory increase in peripheral vascular tone.
**Baraka A, 2012** **[17]**	Rats, 5/6 nephrectomized and controls	Effect of recombinant RNLS infusion on cardiac function in CKD	Partial reversal of LVH, significant reduction in BP; reduction in left ventricular hydroxyproline concentration.
**Lee HT, 2013** **[60]**	Wild-type mice with induced ischemic AKI	Whether administration of RNLS directly protects against ischemic AKI	RNLS ameliorated ischemic AKI by reducing renal tubular necrosis, apoptosis, and inflammation.
**Yin J, 2016** **[54]**	5/6 nephrectomized rats	Role of RNLS in the progression of cardiorenal syndrome after subtotal nephrectomy.	Administration of RNLS reduced proteinuria, glomerular hypertrophy, and interstitial fibrosis after renal ablation. Systemic delivery of RNLS attenuated HT, cardiomyocyte hypertrophy, and cardiac interstitial fibrosis.
**Wu Y, 2017** **[58]**	Rats with unilateral ureteral obstruction Culture of human fibrotic renal interstitial tissue	Effects and mechanisms of RNLS in tubulointerstitial fibrosis	Experimental increased expression of RNLS attenuated renal interstitial fibrosis by suppression of tubular EMT through inhibition of the ERK pathway.
**Wu Y, 2018** **[59]**	Rats with unilateral ureteral obstruction Culture of human fibrotic renal interstitial tissue	Effects and mechanisms of RNLS in tubulointerstitial fibrosis	RNLS protects against tubulointerstitial fibrosis by reducing oxidative stress.
**Wang Y, 2022** **[55]**	RNLS knockout and wild-type mice with CKD	Relation between cardiac expression of RNLS and cardiac remodeling in CKD Effects of RNLS on cardiac remodeling in CKD	Knockout of RNLS aggravated cardiac remodeling in CKD, while RNLS cardiac-specific overexpression significantly reduced LVH and cardiac fibrosis induced by CKD.
**Guo X, 2022** **[61]**	Mice	Whether kidney-targeted delivery of RNLS might prevent cisplatin-induced CKD	RNLS agonist protects against cisplatin-induced CKD by decreasing cell death and improving the viability of the renal proximal tubule

Legend. RNLS—renalase; HR—heart rate; BP—blood pressure; CKD—chronic kidney disease; LVH—left ventricular hypertrophy; AKI—acute kidney injury; HT—hypertension; EMT—epithelial–mesenchymal transition; ERK pathway—extracellular regulated protein kinases.

## 4. Conclusions

Studies performed thus far suggest that renalase could be an important player in the pathogenesis of CKD and could even become a valuable therapeutic tool for preventing CKD progression and related cardiovascular disease. Yet, 20 years after its discovery, renalase remains in the stage of promise. Unknowns regarding its exact mechanisms of action and interaction with other mediators, as well as the lack of a standardized method for measuring serum levels, represent the main barriers for renalase to be used in the therapeutic armamentarium of CKD. Continuing research is needed in this field to ameliorate the poor prognosis of CKD patients.
